# Neural population activity for memory: properties, computations, and codes

**DOI:** 10.1016/j.neuron.2025.11.007

**Published:** 2026-02

**Authors:** David Dupret, Stefano Fusi, Stefano Panzeri

**Affiliations:** 1 Medical Research Council Brain Network Dynamics Unit, Nuffield Department of Clinical Neurosciences, University of Oxford, Oxford, United Kingdom; 2 Mortimer B. Zuckerman Mind Brain Behavior Institute, Department of Neuroscience, Kavli Institute for Brain Science, Columbia University, New York, NY, USA; 3 Institute for Neural Information Processing, University Medical Center Hamburg-Eppendorf (UKE), Hamburg, Germany

## Abstract

The brain’s memory function involves patterns of neural population spiking activity, shaped by experience and recurring over time. These neural population patterns are typically studied with respect to the three stages of acquisition, retention, and retrieval. Despite intensive investigation, the relationship between features of population activity and the properties, computations, and codes for memory remains elusive. In this Perspective, we synthetize recent advances in the study of memory from the viewpoint of brain network physiology, aiming for a comprehensive mapping between the properties and computations of memory and the features of population activity codes. We propose that brain memory circuits implement trade-offs between conflicting demands on population codes. We anticipate that an important challenge for both discovery and translational neuroscience of memory is to study these trade-offs, delineating a safe zone in the population activity space where neuronal circuits operate efficiently.

## Introduction

Memory refers to the acquisition and retention of information related to life experience, the recall of which can affect behavior. Dysfunctional memories are both consequences and causes in virtually all brain disorders, from those characterized by memory loss, such as Alzheimer’s disease, to those involving intensified memories, such as those paired with drug use. Despite its apparent simplicity, the question ‘*How does memory work?*’ remains challenging, yielding numerous scientific investigations and scholastic discussions at the nexus of brain and behavior.

Mnemonic information derives from an interaction with the external world (e.g., remembering a discussion) but can also be linked to the internal world (e.g., remembering a dream). Behaviorists and psychologists have long proposed various classifications to capture the diversity of memories, focusing on the contents, durations, and constructs to interpret observable behavioral readouts ^1,2^. Important biological underpinnings of memory have been described at the levels of molecules, dendrites, synapses, and neurons. A memory is commonly defined in terms of a collection of synaptic weights or of a population of recruited cells. Here, we explore the topic of memory from the perspective of brain network physiology, focusing on neural patterns of spiking activity that emerge through experience and exhibit persistence or functional continuity over time, even if their original version may drift. We focus on how the functional properties of memory arise from those of the underlying activity patterns, which implement specific neural computations. The brain’s memory function can handle many distinct computations, for example, from initial encoding to later updating. Though these operations are all necessary, they may be partly conflicting and difficult to reconcile, as for example when balancing stability and plasticity of neural traces. Findings and concepts on how neural population activity serves memory are blooming ^3–21^. Past ideas (e.g., engrams) are being revisited, and new ones (e.g., geometry of population representations) are being explored. With these, an emerging picture is that understanding the complexity of memory will require considering many features of neural population activity at once. These features interact in complex or even seemingly conflicting ways, reflecting the multifaceted nature of memory. This exciting progress calls for the need to increase our conceptual understanding of the contributions of each population code feature and how these features interact.

To stimulate progress in this direction, we synthetize recent advances in the field of brain network physiology, where recordings, manipulations, and computational tools are paving the way towards a comprehensive understanding of memory. We first discuss memory properties that derive from population activity patterns monitored with cellular resolution using, e.g., multi-channel extracellular electrodes or two-photon calcium imaging. We relate these properties to specific computations performed using different features of neural population codes. In doing so, we propose that brain circuits for memory trade off population activity pattern properties that support computations with often competing benefits and requirements. We consider how neural population codes could achieve optimal tradeoffs between competing functions, including setting values of neural code features that may optimally balance competing demands and using parallel processing channels across space or time. We propose a set of behavioral, neural, and analytical approaches that could be used to determine population-level tradeoffs in memory properties, computations, and codes.

### Functional properties and neural computations of activity patterns for memory

**Figure 1:**
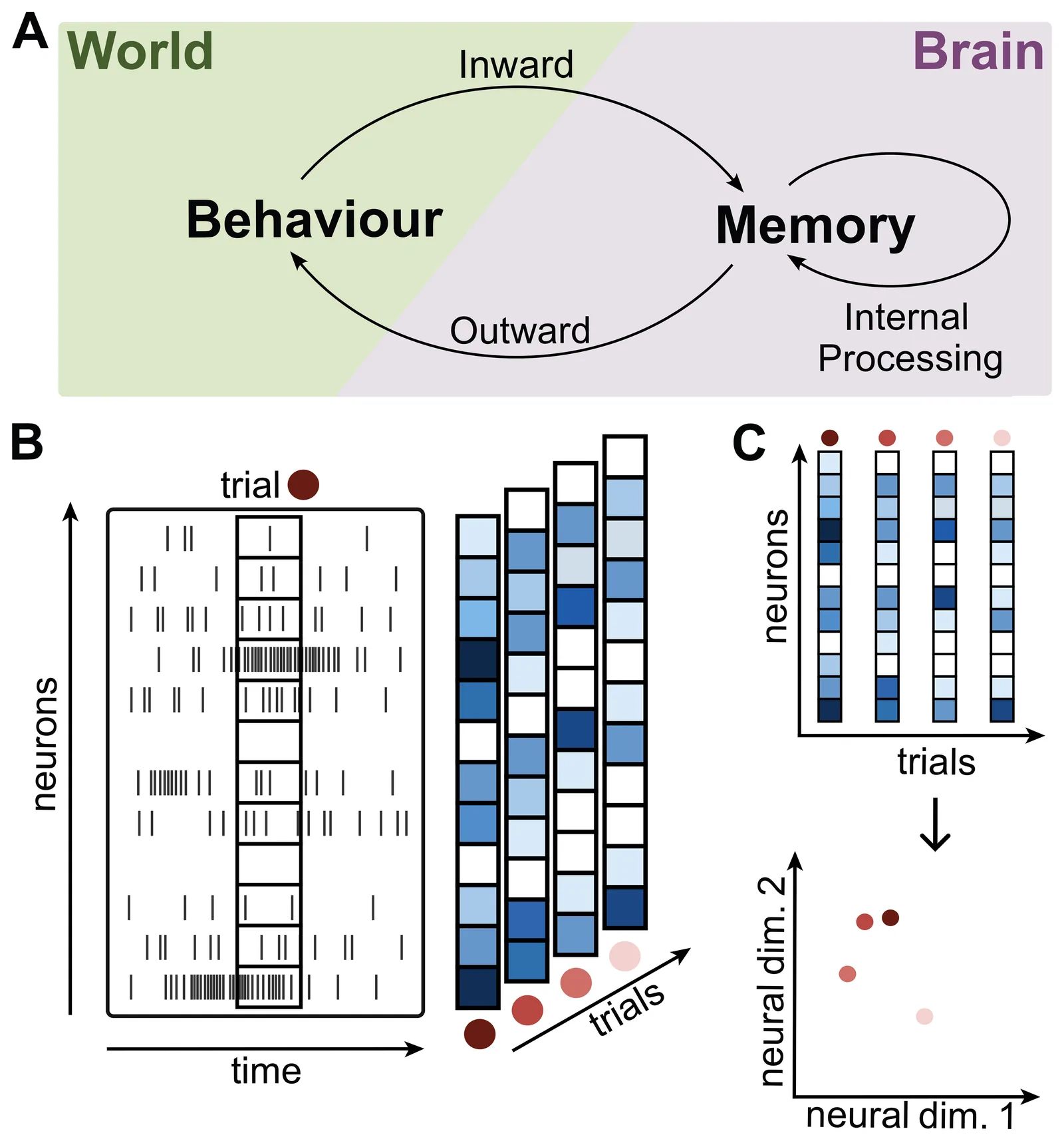
Mnemonic information flow and population activity patterns. **(A)** The inward and outward flows of mnemonic information. **(B)** The spiking activity of the neurons recruited for memory at a given time instantiates a population firing vector made of the firing rate of each neuron at any given time in any given trial. The space of the recorded activity can be described as the set of population vectors expressed across time points and trials and is thus a 3rd-order tensor defined over the neuron-axis spanning the recruited neurons, the time-axis describing the firing changes over time within a trial; and the trial-axis spanning changes across trials. **(C)** The population vectors can be decomposed for further analyses.

To examine the properties of memory activity patterns and their computations, we structure this section according to two directions of information flow (Figure 1A). First, the “inward flow,” through which information from the world gets into memory. Next, the “outward flow,” through which, in turn, mnemonic information is expressed.

#### The inward flow of mnemonic information

During the initial stage of acquisition, the brain encodes the information gathered through behavioral experience into a format usable within the neuronal space. The encoding of the components of a memory (e.g., a location paired with a sound and a reward) relies on the recruitability of the neurons that are available and tuned to represent the incoming information. The spiking of recruited neurons yields a memory activity pattern, which can be quantified as a population activity vector (Figure 1B). This population vector will evolve over time, for instance flickering between old and new memory representations as the current event is compared with previous ones ^22–24^. It will also evolve over trials (e.g., recall instances), for example gradually drifting as the memory is altered across experiences ^12,13,25^. The obtained set of population vectors can be reduced in dimensionality by projecting them onto different axes, such as axes of principal variance (Figure 1C).

Extensive work has uncovered diverse molecular and cellular mechanisms for memory encoding, involving important synaptic-level changes (e.g., receptor trafficking, protein synthesis, and structural plasticity^26–30^). While not the focus of this *Perspective*, these local modifications likely shape mnemonic population codes. Hebbian plasticity is thought to underlie many forms of memory, particularly those formed when stimuli, actions, and outcomes are tightly linked in time such as in classical conditioning. For memories involving associations that unfold over longer timescales, behavioural time scale plasticity (BTSP) ^31,32^, which operates over seconds and with minimal repetition, provides a mechanism for linking experience-driven changes to prolonged neuronal population responses. Many studies have also explored the neuronal allocation to a memory “trace” ^33^, notably by tracking the expression of immediate-early genes (e.g., *c-Fos*) across trials ^34–37^. Terms such as “engram,” “assembly,” or “ensemble” ^38–40^ refer to the set of neurons forming the “neuronal content” of a memory. The spatio-temporal organization of the corresponding activity pattern gained through experience involves various forms of firing tuning, notably including spatially-tuned (place and grid) ^41^ and time ^42^ cells. The expression of these response properties and their remapping reflect neuronal representations of information encoded in memory.

**Figure 2:**
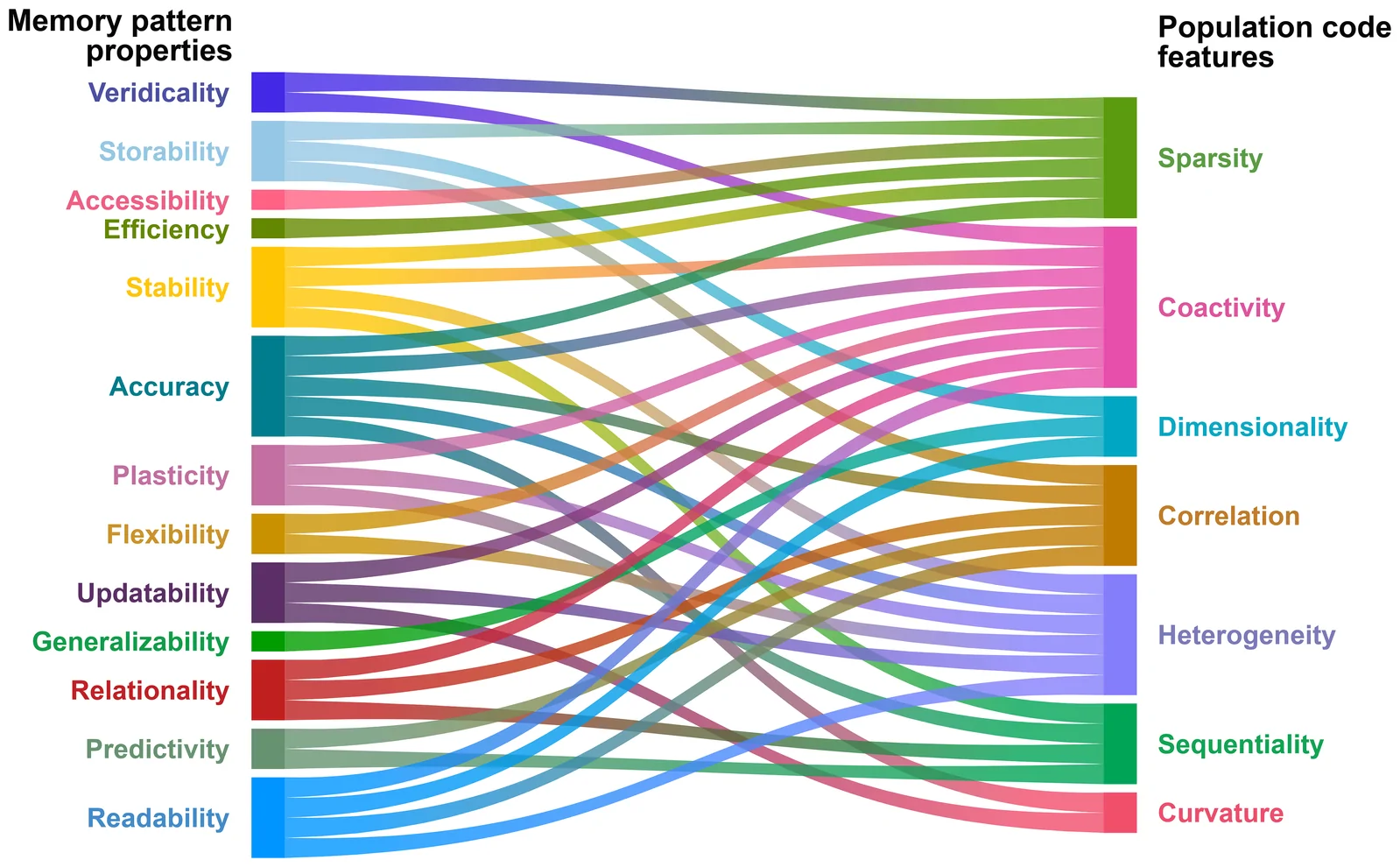
Putative mapping of the properties of memory activity patterns onto features of neural population codes. A definition of each term is reported in the Glossary.

Primary properties of a memory activity pattern are summarized in Figure 2. One such property is the veridicality of the encoded information. This refers to how closely the neural representation reflects the external world. This correspondence is essential to understanding the world (i.e., construct a generative model of sensory experiences), making accurate predictions, and interacting efficiently with the environment. A veridical memory reflects the external world at past times.

Recruiting a too-large set of neurons to comprehensively represent all the items composing an experience would strain the circuit’s ability to store and later access a too-detail-rich memory. Thus, memory neural population patterns need careful design to be retainable over time (storability) and to be retrievable from storage (accessibility). The continual acquisition of memories over a lifetime requires a high storage capacity. Like in computers, storage capacity in the brain can be measured in bits ^43^. Neuronal circuits do not store spikes, but their information content. This is thought to take primarily the form of synaptic strength ^44,45^. For accessibility, a circuit may recruit neurons to retain an index of the brain areas directly representing memory items ^46,47^, akin to pointers storing the address of variables in computer programming. Given the high metabolic demands of the brain, an activity pattern would also need metabolic efficiency, that is minimal expenditure of metabolism for its operations.

#### The outward flow for behavioral expression

The information getting into memory undergoes further internal processing to eventually be retrieved and influence behavior (Figure 1A). Following the initial experience, the established representation is consolidated for longer term storage ^48^. Hebb proposed that memory is initially encoded in “reverberating circuits” ^49^. In the absence of interruptions, this reverberation stabilizes the acquired activity pattern. Subsequently, interrupting this reverberation would no longer compromise patterns due to their strengthening. In Buzsáki’s two-stage model ^50^, supported by a large body of work, the learning-induced changes in hippocampal population activity reverberate offline in sharp wave-ripple events during sleep and rest for consolidation. Stability may thus constitute the core property of a stored memory, ensuring that encoded information resists time and interference for later retrieval. Both assessing immediate-early gene expression across distinct time points and recording neuronal ensembles across multiple behavioral sessions support this notion. Activity-dependent tagging of c-Fos–active neurons showed that learning and retrieval of a fear memory activate the same amygdala neurons ^35^. Silencing “engram” cells in the amygdala or the hippocampus impairs retrieval ^36,51,52^. Their artificial activation induces retrieval ^37,53–55^. Under stable experimental conditions, the spatially tuned activity of the subset of hippocampal neurons mapping a given environment is consistently observed across repeated exposures ^41^. Similarly, hippocampal population patterns formed by coactive neurons in spatially tuned local assemblies reoccur during re-exposure to the same enclosure ^56,57^. Closed-loop silencing of hippocampal ripples during sleep or rest destabilizes subsequent reinstatement of newly-acquired patterns and memory retrieval ^58–60^. Yet, stability over longer time periods may not rely on the exact same cells that originally encoded the memory. Manipulations promoting offline strengthening of new ensembles promote pattern reinstatement and memory performance ^61–63^.

Another important property of a memory pattern is high representation accuracy. In operational terms, memory accuracy refers to how well the memory content represented in neural population activity can be decoded from the population activity pattern when it is reinstated in individual recall trials. Accuracy requires two computations. One is pattern separation, defined as the ability to generate different neural patterns for distinct memories.^64^ The other is pattern precision, defined as the ability to reliably repeat, on a recall-by-recall basis, the memory activity pattern. In principle, a memory pattern exhibits both veridicality and accuracy for its information to be correct and repeatable. Psychologists have however long recognized that memory is prone to distortions, suggesting that it essentially operates as a constructive process rather than simply reproducing past experiences ^65,66^. From an electrophysiological standpoint, how to assess the extent to which a stored and retrieved activity pattern for memory faithfully matches the physical truth of what has been originally experienced and encoded is challenging.

Memory can undergo various alterations. It is more a constant reconstruction than a faithful reproduction.^65–68^ Upon recall, a memory re-enters a labile state, permitting the integration of additional information well after the original experience ^69^. The expression strength of recalled hippocampal patterns tends to be weaker despite consistent surrounding conditions ^60^. Changes in pattern separation and precision across instances of memory expression could underpin a representational drift ^12,13,25,70–73^, with patterns moving away from their originally coded version. Artificially activating in one context the neurons associated with another context creates a hybrid memory trace ^74^. Selective silencing of neurons recruited during the formation of a contextual memory allows previously quiet neurons to emerge for the computation of an alternative representation ^36^. These observations suggest that a memory activity pattern faces a trade-off between maintaining stability to function as a ‘trace,’ versus allowing plasticity and flexibility to respond to an ever-changing environment. By plasticity, we refer to the ability of a memory activity pattern to undergo a transformative and enduring change in response to an external influence. Flexibility, on the other hand, refers to the ability of a memory pattern to undergo temporary adjustment to immediate needs. To continually support adaptation, memory also needs updatability, editing past information, and incorporating new information to enhance functionality. Like plasticity and flexibility, updatability can be a response to external factors, but it primarily reflects an internal improvement process, for example to support the ability to add task context to an existing motor memory ^14^. A circuit hosting a memory pattern might later prune what is unimportant or inaccurate. All these changes would indicate that memory activity patterns allow rheostasis ^75^.

Another property of memory is generalizability. It involves the neural computation of pattern generalization: memory patterns should allow for representations in a format that is abstract enough so that the information content can be retrieved independently of the original context and used across situations. For example, for social memory, the neural circuit must generalize to detect whether an individual is familiar across contexts, independently of the individual’s identity or their location ^5^. Memory can also link multiple population patterns to form functional associations. This computation enables two properties. One is relationality, that is establishing relationships between memory patterns that have not been directly associated but are temporally or logically related. For example, this may serve inferential reasoning ^76^. Another is predictivity of future events. For example, sequentially organized hippocampal firing activity to instantiate a predictive model of optimal behavioral trajectories serving memory-guided navigation ^15^.

For translation into a readout, neurons representing memory information will not only need to retrieve the appropriate pattern but also make it readable downstream. This readability requires tailoring the activity pattern so that receiver neurons can read it effectively ^77^. In that sense, the accuracy of a memory representation is what an ideal observer (operationally, a well-trained machine-learning algorithm) can extract from the retrieved activity pattern. Readability refers instead to how well the represented information is received downstream. While having a higher representation accuracy would in general be beneficial to downstream readability, accuracy and readability are partly dissociated. For example, a pattern with lower accuracy may have higher readability. This could happen because it can be read out by simpler operations that would be realistically accessible by neurons (e.g., linear downstream decoding ^78^) or because it is expressed in a format that promotes biophysical downstream propagation (e.g., organizing spikes by increasing correlations) ^38^. By combining multiple properties, from accuracy to stability to readability, a memory activity pattern eventually gains robustness.

### Features of population activity in neural codes for memory

Each of the memory properties and associated computations listed above is performed through specific **neural population codes**, defined as features of population activity that set the format of mnemonic information. Does each computation rely on a one-to-one mapping with a single neural code? Or does each computation involve a one-to-many mapping with multiple codes? Does a single neural code impact one or multiple memory properties? Is there an optimal structure of neural codes to serve a specific property?

Here we consider different features of neural population codes and how they can affect memory computations and properties (Figure 2). We focus on the features of population firing vectors that report, at a given time, the number of spikes discharged by each neuron. These population vectors can be monitored with cellular resolution using spike-sorted signals from electrophysiological ensemble recording or from calcium imaging ^79,80^. This is a relevant level of organization to focus on, as brain computations are implemented through interactions between neurons ^81^. Many studies have also emphasized the importance of network rhythms (and their coordination across spatial and temporal scales), which are notably detected in fluctuations of the Local Field Potentials (LFPs) ^82–85^. We will highlight below the importance of this level of organization and the insights gained by focusing not on one but on multiple levels. In this Section, we examine how the organization of neural population vectors influences memory properties and computations, considering three axes (i.e., a 3rd-order tensor) along which population vector features can vary: *across neurons, across time* points, and *across trials* (Figure 1B).

#### The across-neuron axis

**Figure 3:**
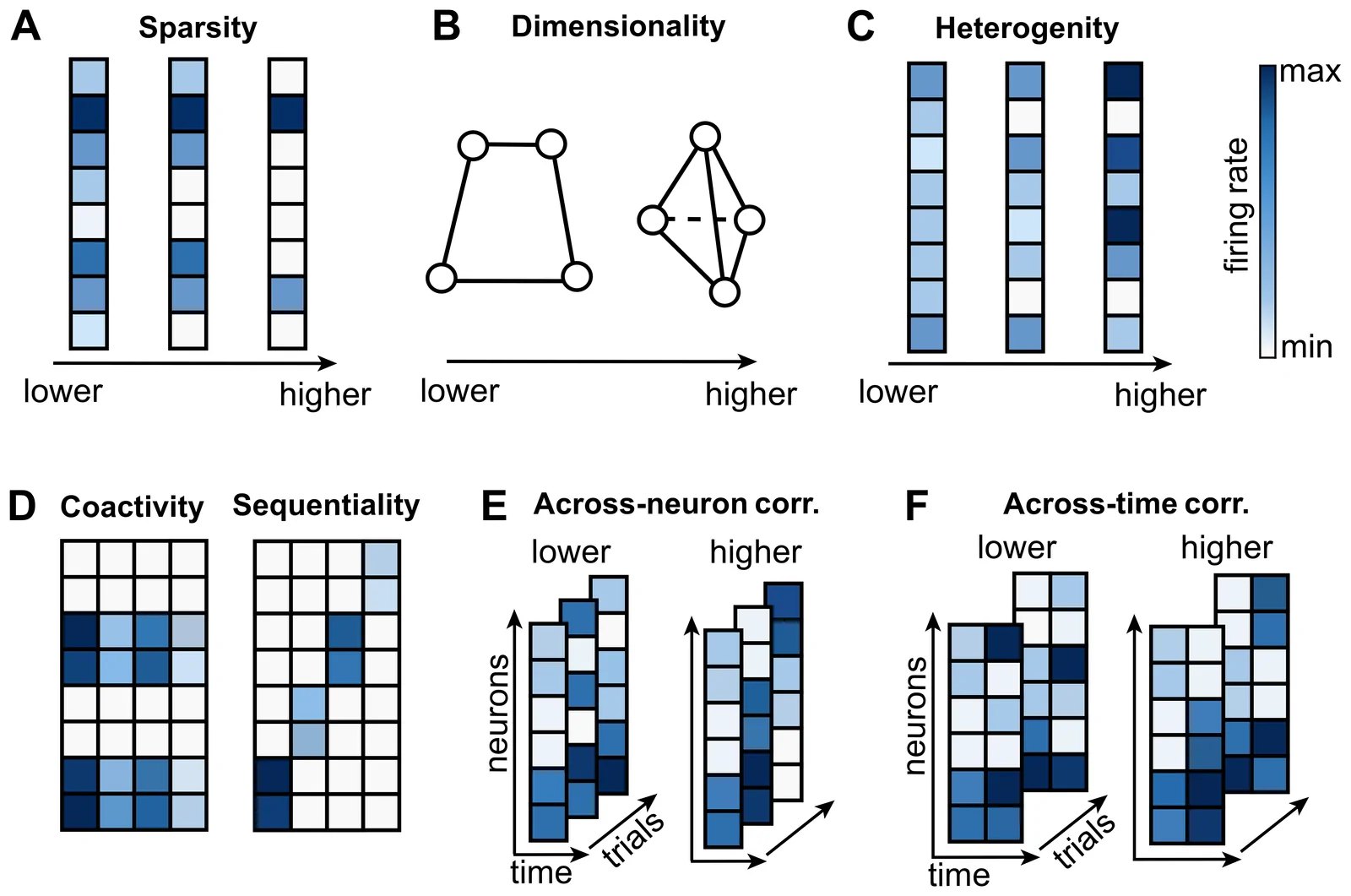
Illustration of population code features. The nature of the feature is illustrated by comparison of cartoons of population activity drawn with higher or lower values of the considered feature. **(A**,**C-F):** Sparsity, heterogeneity, coactivity, sequential activity, across-neuron and across-time correlations are illustrated with cartoons of population vectors, with each square representing the firing response (whose strength is plotted using the color-scale on the top row, right) of a neuron at a given time point. The across-neuron correlation illustration shows adjacent neurons, that when more correlated, show concomitant increased or decreased activity during the same trial. The across-time correlation shows that the two correlated population vectors maintain a similar shape of the population vector across time. **(B)** Dimensionality is illustrated with cartoons in which population vectors of different memory items are dots in a 3D space and lines are used to show whether they line on the same 2-D plane (left) or not (right).

Features that distinguish population vectors along the *across-neuron axis* include the sparsity, the geometry, and the heterogeneity of population activity (Figure 3A-C). Sparsity quantifies the fraction of neurons active at a given time (Figure 3A). (Note that population sparsity differs from the sparsity in the spike train of an individual neuron that corresponds to high kurtosis in its lifetime response distribution ^86^). Theoretical and empirical studies have suggested that population sparsity affects *storability* of activity patterns. Sparse population representations (i.e., with few active neurons) allow for a larger number of memories to be stored without overwriting previous memories because of the reduced interference between stored patterns ^64,87–89^. This holds under the assumption that the neurons active in each representation are evenly spread across memories. However, sparse patterns store fewer bits of information in each memory ^90^. They favor representation accuracy, as they achieve a good trade-off between pattern separation and precision ^78^. As neural activity is metabolically expensive ^91^, sparse representations have high bioenergetic efficiency, with good accuracy at lower metabolic cost and higher amounts of information per spike ^92^. Sparsity may support veridicality by restricting pattern encoding to essential mnemonic items or to compressed memory representations ^93,94^. Finally, sparsity also influences stability by prolonging memory lifetime via synaptic metaplasticity ^95,96^.

**Figure 4:**
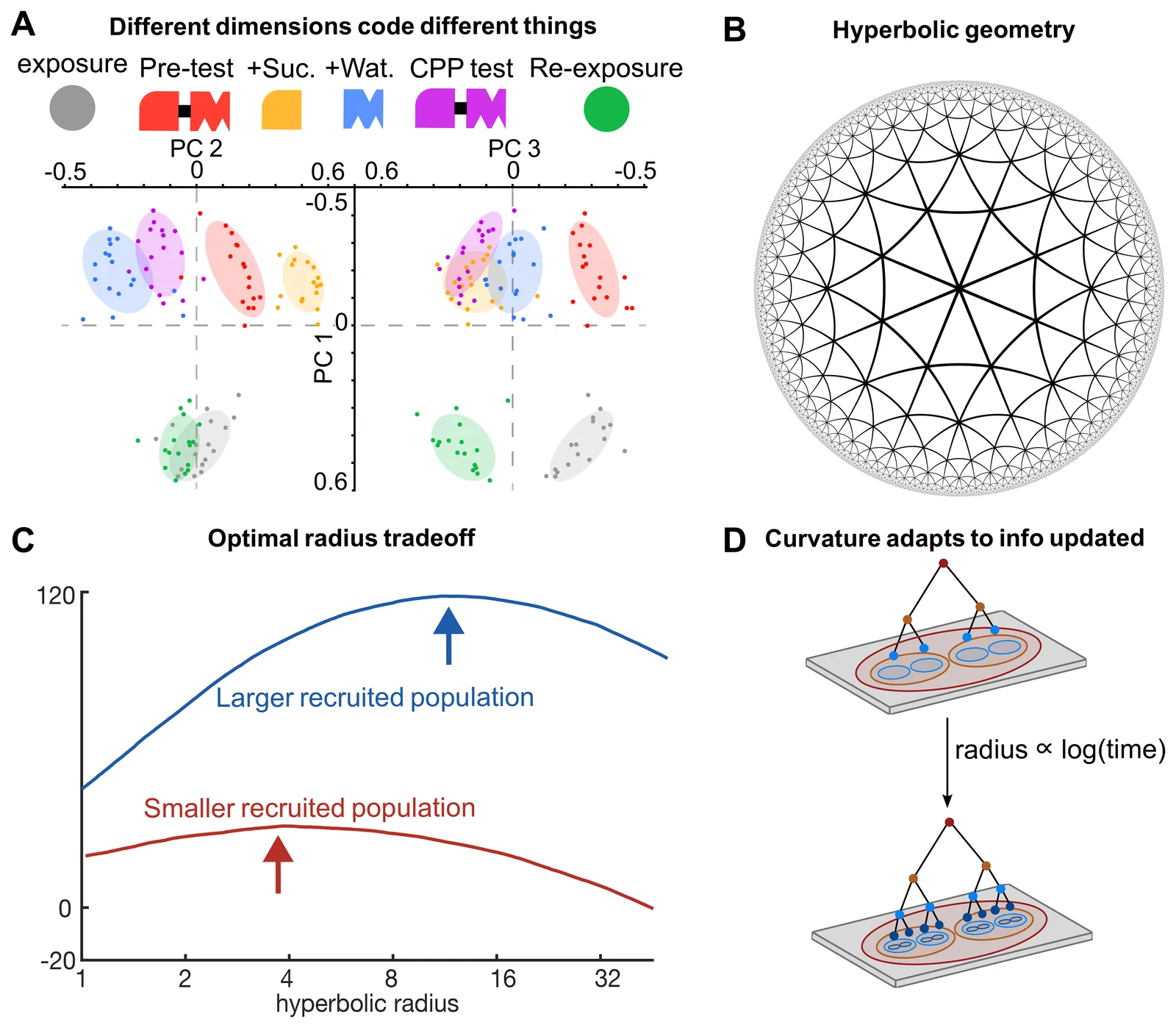
Possible roles of neural activity geometry in population coding **(A)** Example from a memory task (Conditioned Place Preference, CPP) showing that different dimensions (first 3 principal components) of the hippocampal CA1 population activity carry different information. *Top*, Task layout (six color-coded sessions). Each day, mice explored the same familiar enclosure twice: before (exposure) and after (re-exposure) four sessions in the CPP apparatus (pre-test, sucrose +Suc, water +Wat, and test). *Bottom*, Applying principal component analysis to matrices of topological (Riemannian Log-Euclidean) distances between coactivity motifs revealed three axes explaining across-session variance in population activity: PC1 segregated sessions in the familiar enclosure versus those in the CPP apparatus, PC2 segregated the four individual CPP sessions, and PC3 segregated the pre-conditioning versus post-conditioning exploration of the familiar enclosure. Each data point represents one (color-coded) recording mouse session. Re-drawn from *Gava et al*. ^165^ **(B)** Projecting on a flat surface the Poincaré disk (a 2-D space with hyperbolic geometry) highlights the similarity of 2D hyperbolic geometry with hierarchical population representations. Each curve represents the geodesic (the shortest distance path) between two connected points, and all triangles have the same size. Modified from public domain material commons (wikimedia.org/wiki/File:H2-5-4-kis-primal.svg). **(C)** Fisher information about the animal’s position in the physical space represented in population activity for populations of hippocampal neurons encoding space with hyperbolic geometry (considering different sizes of the recruited neural populations). Information values peak at non-zero values of the hyperbolic curvature radius, meaning that representations of the physical space in curved neural spaces are advantageous. Recruiting larger populations increases the available information and shifts the optimal curvature (vertical arrows) toward favoring more curved spaces. **(D)** Curvature and updatability. Top: Illustration of how hyperbolic geometries arise from combining hierarchically populations of place fields with different sizes. Bottom: adding more neurons with small place fields makes the geometry more curved (compare with panel B). In real data, the curvature radius increases proportionally to information about space gained spending time exploring an environment. Panels C and D replotted from *Zhang et al*.^18^

We then consider the geometry of the population activity space hosting neural population patterns. This geometry is defined by the set of distances between the representations to be stored. It can be characterized by its embedding **dimensionality** (Figure 3B), defined as the number of coordinate axes needed to specify the position of the memory representations in the population activity Euclidean space ^97–99^. For example, this dimensionality would be two if the population vectors elicited across different conditions lie on a plane (Figure 3B, left). Representations with low embedding dimensionality limit memory storage capacity, because they have a greater correlation between the activity of different neurons, reducing the amount of different patterns that can be stored ^5^. At the same time, these heightened correlations promote robustness and readability (see below). Representations with higher embedding dimensionality provide higher memory capacity, but reduced generalizability. Therefore, different dimensionalities could satisfy different memory demands by controlling the trade-off between generalizability and storability of memory activity patterns ^5^. The memory storage capacity critically depends on neural noise. For example, elevated noise along the axes connecting the neural representation of one memory to those of other memories limits the ability to retrieve distinct memories. Higher dimensionality formally guarantees higher memory capacity in the absence of noise ^100^. This principle also applies to real-world problems: indeed, support vector machines use effectively non-linear kernels to increase the dimensionality, even with noisy data ^101^. Neural network classifiers also operate in the presence of noise ^102^. A memory circuit may leverage dimensionality for concomitant operations. For instance, the diversity of hippocampal ripples ^16,17,103^ allows the higher-dimensional patterns that flexibly integrate recent firing motifs to co-exist with the lower-dimensional patterns that maintain core motifs of prior firing activity ^103^. During memory processing, hippocampal population spiking activity organizes across multiple complementary axes to represent the different items experienced (Figure 4A) ^104^. Moreover, the geometry of the population activity manifold, even if we embed it in Euclidean spaces to compute population vectors, might be non-flat and non-Euclidean (e.g., a Riemannian manifold). It is further characterized by an intrinsic dimensionality (the minimal number of variables needed to parameterize the manifold). If the population activity manifold is not flat, additional geometric features, beyond dimensionality, are needed to characterize it. One such feature is the **curvature** radius ^18^. Curved spaces can result from attractor dynamics ^105,106^. Curvature influences information representations. Hippocampal codes for spatial position live in a population space with hyperbolic geometry, which is parameterized by its curvature radius and arises by combining hierarchically organized neurons with place fields of different sizes. When larger place fields are introduced—spanning multiple smaller ones—the representation becomes hierarchical and curves the neural activity space (Figure 4B) ^18^. Representation accuracy in hyperbolic spaces is optimized by intermediate non-zero optimal curvature values that depend on the size of the recruited population (Figure 4C), with larger recruited populations being able to encode position with higher accuracy and having optimal representations at more curved neural population spaces (Figure 4C), which include neurons with smaller place fields (Figure 4D). Varying the curvature radius of a population space could serve memory updating, as acquiring more information over time expands the radius towards curvature levels optimal for representing new information (Figure 4D) ^18^.

The heterogeneity or diversity of single neuron tuning in memory circuits (Figure 3C) profoundly shapes information computations. The accuracy of information representations improves for populations with heterogeneous tuning properties compared to populations with homogeneous tuning properties, as heterogeneous representations are less damaged by correlated noise ^107^. Recent research is unveiling a significant heterogeneity among CA1 principal cells, which form two parallel channels with distinct properties ^108–112^. In rodents, CA1 principal cells with somatic location in the deep pyramidal sublayer exhibit higher rate and more rigid activity; their superficial counterparts show lower firing rate and more plastic activity ^113–120^. Their functional mixing can balance the requirement for stability, plasticity, flexibility, and updatability in memory patterns. During acquisition, higher-activity neurons could support memory accuracy due to their rapid engagement in ongoing behavior. Gradual engagement of lower-activity neurons could strongly influence the trade-off between stability and plasticity, flexibility, and updatability. The minority of higher-activity principal cells instantiates a pre-structured scaffold onto which additional (lower-activity) neurons contribute to build more complex, higher-capacity memories ^9,103^.

#### The across-time-points axis

Population firing vectors can be distinguished by how they evolve *across time* points. Two major cases are observed: simultaneous activity (coactivations) and sequential activity (sequences) of neurons (Figure 3D). Both have been reported for many brain functions, including beyond memory ^92,121^. In the hippocampus, they have been documented for principal cells, e.g. by assessing the temporal organization of spiking activity with respect to place fields and network oscillations (e.g., theta cycles during exploration and ripple events in sleep/rest) ^38^.

Coactivity refers to the transient coincidental firing of groups of neurons within short (few tens of ms) windows. Coactivation can be related to similar tuning of neural activity to external variables, or to across-neuron correlations (see below). Higher coactivation among neurons with similar firing tuning ^57,60^ allows robust information transmission, helping with downstream readability for a potent influence on behavior ^20,56^. This could occur because more coincident spikes aid readout by receiver neurons with short integration time constants or some supra-linear dendritic integration of near coincident inputs. Coactivating many neurons thus serves memory robustness and readability. Transient coactivation of different neuronal ensembles organized across different time epochs supports memory flexibility by means of moment-by-moment expression of distinct population patterns ^22–24,122^. Coactivity nested in hippocampal ripples during post-exploration sleep and rest instantiates the offline reactivation of waking population patterns ^58–60,122,123^, supporting memory representation stability and plasticity. The topology of population coactivity, that is the organization of the firing relationships between neurons (which can be conceptualized as “nodes” in a network), also contributes to memory properties. High clustering and short path lengths between nodes in a network produce a small-world topology, allowing effective coexistence of local and global computations ^124^. Higher clustering coefficient and longer path lengths feature more stable (even rigid) memory representations ^20,125^. Coactivity computed for learned contingencies also supports relationality, allowing an emergent population code to represent associations that single neurons do not represent individually ^15,57^. Coactivity can support the holistic integration of neuronal representations distributed across brain networks (even those not directly connected; e.g., dorsal hippocampus and amygdala) ^126^. The temporal overlap permitted by coactivity supports both relationality and updatability by allowing a memory pattern to engage neurons that were either previously committed or newly recruited during other experiences. This way, experienced items can be flexibly linked, online or offline, for memory-based inference and the integration of related memories ^21,76,127^. However, coactivating multiple representations can also produce interference, affecting accuracy by compromising pattern separation and precision. Minimizing coactivations to similar variables can help to compute orthogonal representations e.g. when learning tasks with different structures ^10^.

**Sequential activity** refers to neuronal spiking organized to sequentially tile time windows longer than those of coactivity. Sequential activity at multiple timescales is central to hippocampal memory function ^128–132^. During spatial exploration, place cells fire sequentially as the animal samples space. At this behavioral timescale of seconds or longer, sequential activity reflects the ongoing animal’s trajectory. Concomitantly, sequential activity is organized at the faster (100–150 ms) theta timescale, reflecting past, present and future locations and supporting both memory encoding and recall ^132–137^. Sequential activity in theta oscillations is relevant to forming temporal links between mnemonic items, which serve remembering successive events, predicting possible outcomes, and planning future actions ^15,84,135,138–141^. During sleep and rest, internally generated sequential activity can recapitulate waking patterns at an even faster (50–100 ms) pace during hippocampal ripples ^82^. Ripple sequential activity instantiates replay ^142,143^, which supports offline memory stabilization and plasticity ^144,145^. Sequential activity also serves planning and is observed across cortical areas when performing working memory or decision-making during navigation ^62,138,146^. By virtue of tiling longer time windows, relative to coactivity, sequential firing serves predictivity ^15^. Population spiking in the medial entorhinal cortex can even organize into ultraslow sequential activity, which would support relationalit*y* across extended (tens of seconds to minutes) timescales ^8^.

#### The correlated trial-to-trial variability axis

The third conceptual axis is the **correlated trial-to-trial variability** of population activity. We use correlation as a general shorthand to indicate any statistical relationships between variables (i.e., not necessarily linear Pearson correlation). This can take the form of **across-neuron correlations** or **across-time correlations**. The former are defined as correlations between the spike counts of different neurons within the same time bin, whereas the latter are defined as correlations between the population activity of the same neurons at different time bins. In both cases, correlations are computed over trials. For example, when two or more neurons are all more active or less active than average in the same trial, there will be a positive across-neuron correlation (Figure 3E). When fluctuations in population vectors at earlier times correlate with those of the population vectors of the same population at later times within the trial, there will be a positive across-time correlation (Figure 3F). Although across-neuron correlations or across-time correlations are computed along different directions of the population activity space, we expect them to contribute similarly to neural coding ^77^ and will thus be mostly discussed together. Also, such correlations are often computed (e.g. so called noise correlations) using only trials with the same time task condition (e.g. same cue), to discount the effect of spurious covariations due to common tuning of different neurons to the task variables ^147^. The average level of across-neuron correlation influences the number of activity patterns that are expressed and can thus be stored. Increased pattern correlation could yield redundancy in the system for memory robustness ^20^. The higher the average correlation level, the harder it is to orthogonalize population patterns. Both types of correlations across trials change representation accuracy ^147–149^. On theoretical grounds, correlations increase accuracy when they are stimulus modulated, or when the sign of correlations is opposite with respect to the sign of the tuning similarity of the neurons (e.g., positively correlated neurons with dissimilar tuning to the stimuli). Correlations decrease accuracy when they are not stimulus modulated and when the sign of correlations is the same as the sign of the tuning similarity of the neurons (e.g., positively correlated neurons with similar tuning to the stimuli) ^148,149^. In empirical studies, correlations have been found to meet all these theoretical scenarios, either reducing or increasing the accuracy of representations depending on the behavioral task or brain region. In memory circuits, correlations often increase accuracy ^150,151^. In sensory cortices, correlations are weaker and limit encoding less than in association cortices ^77,121,152^. Correlations across neurons and time also enhance the robustness and readability of information by aiding downstream information transmission beyond the coactivation described above ^77,153–155^. More of any given amount of encoded information is transmitted downstream if correlation levels are non-zero. Memory performance may depend on correlation levels with a bell-shaped curve, with intermediate levels trading off advantages and disadvantages for encoding and readout/transmission. Note that correlations are related to, yet distinct from, the above discussed coactivity or sequential activation. For example, neurons that fire in a sequence may do so in two ways depending on whether their activity is correlated or not. They can organize in a chain with a strong across-time correlation, such that the fluctuations in the activation of a neuron at a point of a sequence within a trial correlate with fluctuations of the activity of other neurons that fire later in the sequence within the same trial. Alternatively, they can fire without any across-time correlations, with different neurons in the sequence fluctuating independently ^121^. Similarly, coactivity could derive only from neurons being activated at a similar time because of a similar tuning to the external variables (e.g., visual cue), or it could be made stronger by across-neuron correlations. The former scenario could be created by connectivity between the neurons forming and propagating the sequence ^156^. The latter is when neurons not connected to each other receive different activating inputs at different times. When across-time or across-neuron correlations are added to sequential activity, they can aid relationality and predictivity, as they lengthen the timescales in which information remains consistent over time ^121^.

### Which strategies could allow population codes to optimize memory performance?

The above considerations suggest that certain features of neural codes benefit some memory properties and computations while hindering others. This raises the fundamental question of which strategies neuronal populations could use to optimally perform multiple functions and balance - or trade off - conflicting demands.

**Figure 5:**
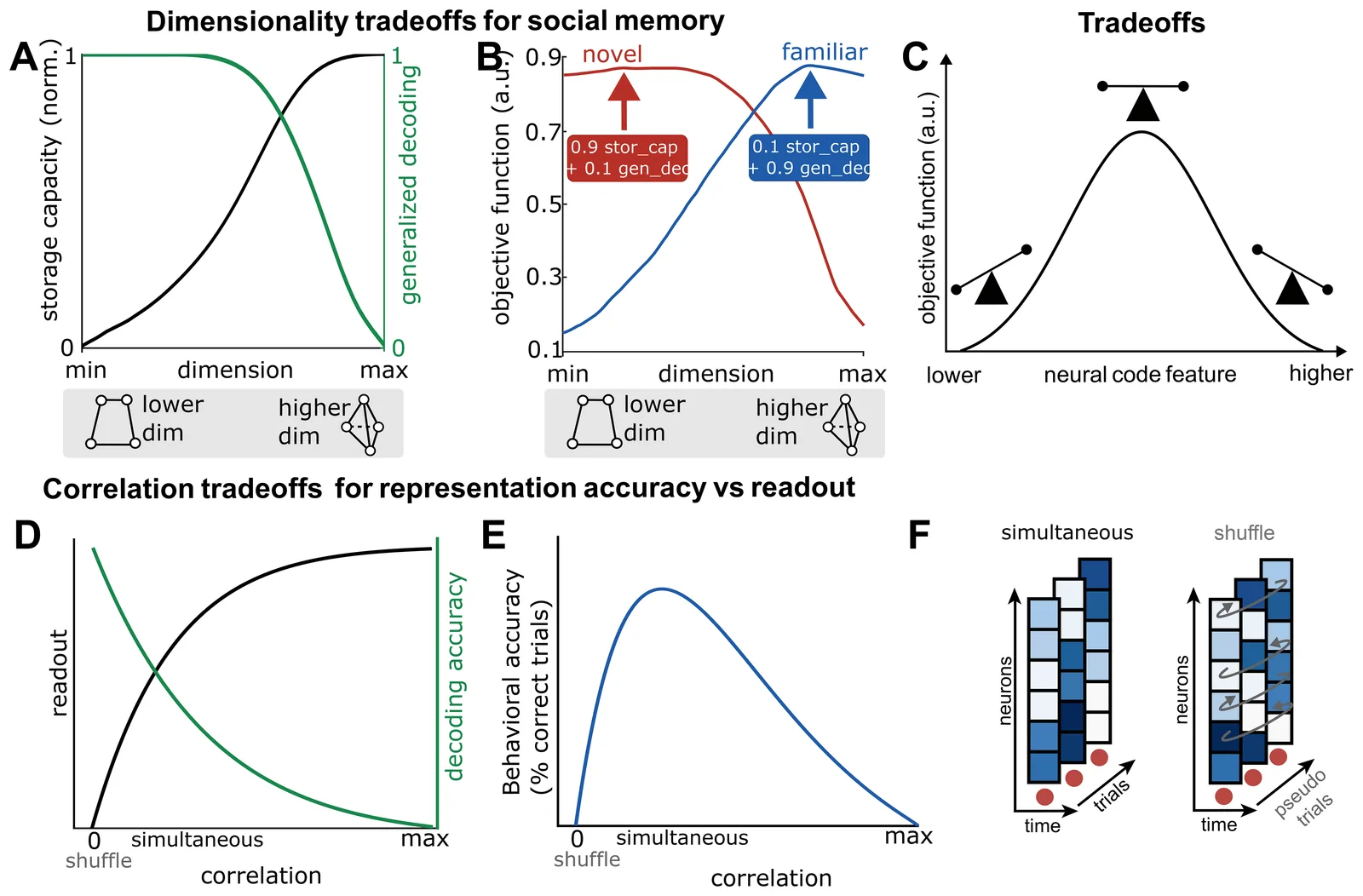
Tradeoffs by optimal balancing. **(A)** Example of how a tradeoff for social memory is understood from competing computations. Low dimensionality favors generalizable neural representations of memories for identities of individuals decodable regardless of context but limits storage capacity. High dimensionality does the opposite. **(B)** An objective function for tradeoff will weigh these two factors. When detecting novel individuals, the objective function (red line) would give higher weight to generalization, and the optimal tradeoff (red arrow) is reached at low dimensionality. When memorizing familiar individuals, the objective function (blue line) would give higher weight to storage capacity and the optimal tradeoff is reached at higher dimensionality (blue arrow). Panels A and B adapted from *Boyle et al*. ^5^ **(C)** Sketch of an objective function of a neural code feature for regulating tradeoffs. The objective function on the y-axis is a weighted sum of different objective functions which correspond to different conflicting demands of the memory task. **(D)** Illustration of a possible tradeoff between representation accuracy and downstream information readout involving correlations. Higher correlation levels favor the downstream transmission and readout of information but may decrease the decoding accuracy of the memory representation. **(E)** Under the conditions of panel D, behavioral accuracy (% of correct trials) may be described with an objective function that trades off accuracy vs readout. It may thus be optimized at intermediate correlation levels, such as those observed in the simultaneously recorded data rather than at zero correlations levels such as those created with trial shuffling. **(F)** The effect of correlation on representation accuracy may be computed from data with analytical methods, by comparing the performance of decoders trained on the real simultaneously recorded population activity vector with that obtained on pseudo-population vectors created randomly shuffling activity of neurons across trials recalling the same memory item.

The first possible strategy is that a neural code feature is designed to simultaneously engage in different operations. In this case, the values of this code feature are chosen to optimally balance different needs and possibly competing constraints. This optimal balancing can be conceptualized as assigning a relative benefit (or cost) to each competing demand and optimizing the obtained weighted objective function. The identity of the objective functions that would optimize each memory demand remains to be identified. In some cases, these can be conceptualized by considering that such functions result from composite computations and studying them in models. For example, social memory requires both memorizing the identity of new individuals across contexts (hence generalization) and storing detailed memories of familiar individuals (hence high storage capacity). These computations may be partly competing; they are pulled in different directions in neural network models when changing the dimensionality of the stored patterns ^5^. Lower dimensionality of stored patterns favors representations of identity that generalize to different contexts, whereas higher dimensionality favors storage capacity (Figure 5A). Situations such as detecting rapidly novel individuals would require an objective function with a higher weight assigned to generalizable representations, with an optimal trade-off reached for low dimensionality (Figure 5B). Situations such as storing memories of familiar individuals may require an objective function with a higher weight assigned to storage which the optimal trade-off reached for higher dimensions (Figure 5B). In both such examples, the objective function weighting the competing constraints has an optimal point at intermediate (non-zero, non-infinite) values of the considered feature (in this case the dimensionality). In general, an objective function weighting competing constraints will be expected to have a maximum at a certain intermediate value of the feature, which represents the optimal balance between the two constraints (Figure 5C). The relative importance of competing needs may change depending on various parameters, such as the memory load and the behavioral contingencies. This may later require updating neural activity feature values to continue supporting the trade-off optimality under new conditions. Similar considerations may apply to many scenarios. Trade-offs between generalizable and detail-rich specific memories are a recurring theme in contextual memory, including context-fear generalization ^157–159^. Whether a single set of neural features underlies all such trade-offs remains unclear. However, theoretical work ^78,160^ has examined the computational implications of the representational geometry of contextual memories. When the information about context, and the information shared across multiple contexts (e.g. the identity of an object that appears in different contexts) are represented in distinct neuronal populations, generalization can be readily achieved with a readout mechanism that can simply selectively ignore one population. However, in this representation the activity patterns representing the distinct memories are strongly correlated, which in turn decreases the dimensionality of the memory space and limits storage capacity. More broadly, the storage and retrieval capacity of memory systems is shaped by neural features such as sparsity and inter-pattern correlations. Since forgetting is essential for limiting information accumulation in memory, it too may be subject to trade-offs. In neural network models, memory overload can lead to sudden blackouts of all memories (blackout catastrophe ^102^). Even when memory is not overloaded, catastrophic forgetting can be induced by temporal correlations between the stored memory patterns or different learning samples ^161^. Active forgetting can also contribute to make these trade-offs more favourable ^162^. Notably, active engram forgetting has been proposed as a mechanism for selectively discarding information, freeing capacity for more salient, recent, or relevant memories ^163,164^. Finally, correlation levels may also mediate a trade-off between accuracy and readability of memory patterns. The accuracy of memory representations in neural populations may be decreased by higher correlation levels that instead boost the downstream readout (Figure 5D). Recalling information to generate appropriate behavioral outputs may thus involve a tradeoff between representation accuracy and readability. The tradeoff may be optimized at intermediate correlation levels (Figure 5E,F).

**Figure 6:**
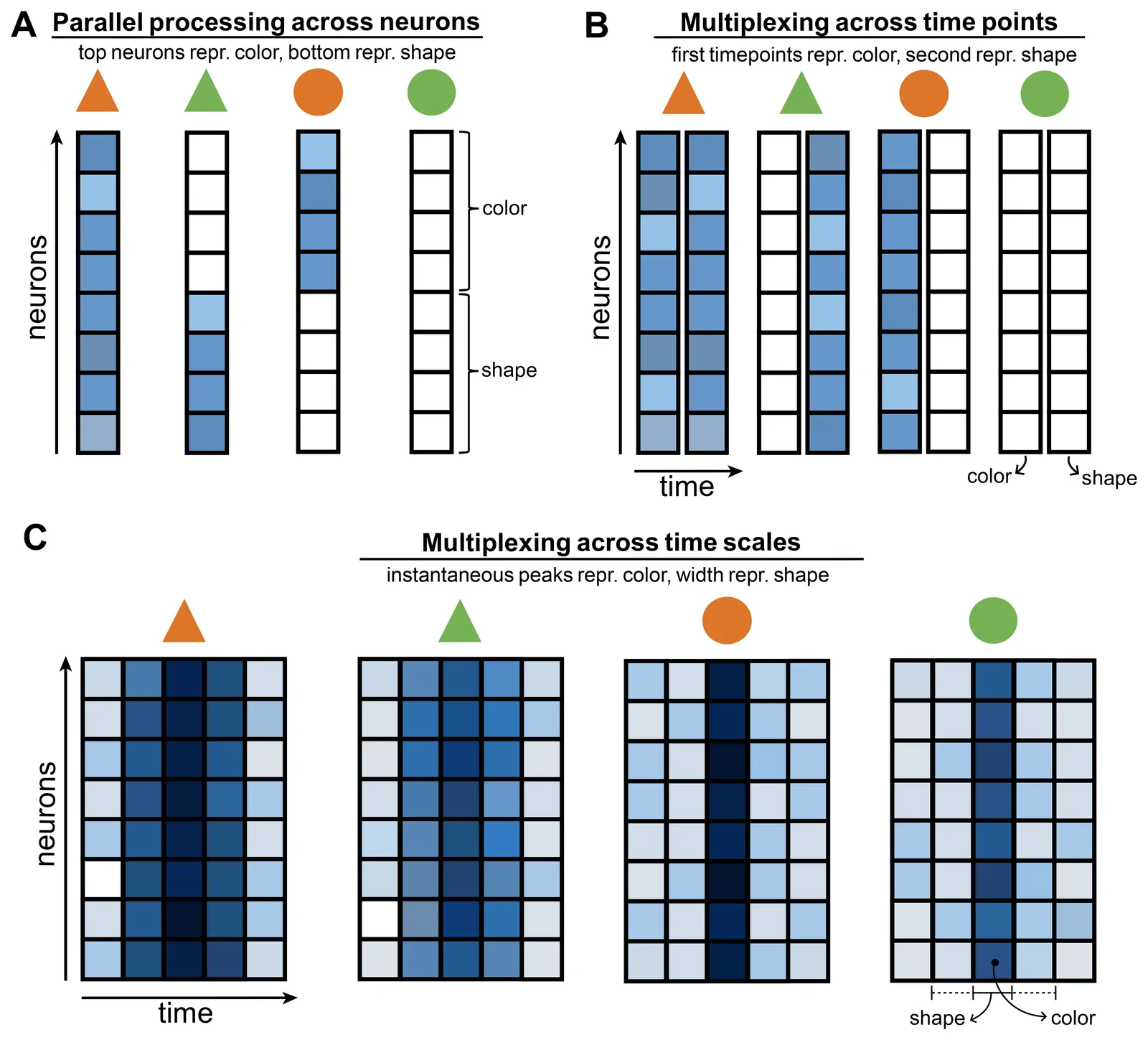
Strategies of population coding for performing multiple functions. Three ways in which population activity could help performing multiple functions. We illustrate these with cartoons of population vectors elicited with four possible memories (i.e., four memory recall trial types; indicated at the bottom) with a 2-bit content (shape and color of an object). In these population vectors, each square represents the firing response of a neuron at a given time. **(A)** Parallel processing across neurons. A different computation is assigned to different sets of neurons that are all active at the same time. The top neurons represent color by elevating firing to orange and decreasing to green, but do not represent shape. The bottom neurons represent shape by elevating firing to triangle and decreasing to circle regardless of color. **(B)** Across-time-points multiplexing. A single neural population is assigned different computations to be performed at different time points. The neurons represent color elevating firing to orange and decreasing to green at an earlier time. They represent shape elevating firing to triangles and decreasing firing to circles at a later time **(C)** Across-timescales multiplexing. A population is assigned multiple computations at the same time point, with different computations assigned to slower vs faster timescales. The neurons represent color by the instantaneous level of peak activity (a higher level of it represents orange and a lower level represents green) and represent shape by how long the activity lasts (longer responses represent triangles, shorter responses represent circles).

The second strategy would be that the brain resolves competing demands using parallel processing to assign specific computations to different units of neural activity. We envisage three such solutions (Figure 6). In the first solution, which we term *across-neuron (i.e., spatial) parallel processing*, a memory circuit assigns different computational tasks to distinct components of activity within a population (Figure 6A). These components or “modules” can either be separate subpopulations or also orthogonal activity components simultaneously present within a population ^165,166^, and would be active at the same time, allowing instantaneous parallel processing channels. For instance, novel sequences of place cell firing combine the complementary contributions of a set of rigid fast-firing pyramidal neurons showing low spatial specificity with a set of plastic slow-firing pyramidal cells gaining high place specificity during exploration, increased response to hippocampal ripples, and heightened burstiness and temporal coactivity ^115^. Such division of labor may reflect an information-theoretic optimization strategy ^167^, allowing contextual signals to dynamically modulate circuit function (e.g., dynamically biasing specific subpopulations toward encoding or retrieval modes) while preserving high-rate, rigid neurons for stable high-fidelity encoding.

The other two solutions parallelize computations using the time domain. With an *across-time-points multiplexing*, a single population performs different computations at different time points (Figure 6B). For instance, the same population of neurons in the primate inferotemporal and perirhinal cortex represent both the percept and memory of faces using a distinct long-latency code for temporal multiplexing ^6^. During spatial exploration, the firing activity of principal cells in the rodent hippocampus is parsed over theta oscillations to transiently support memory encoding or retrieval ^122,168,169^. With an *across-timescales multiplexing*, a single population performs multiple computations at the same time, but with some computation performed over faster-timescale activity and other computations performed over slower-timescale activity (Figure 6C) ^170^.

**Figure 7:**
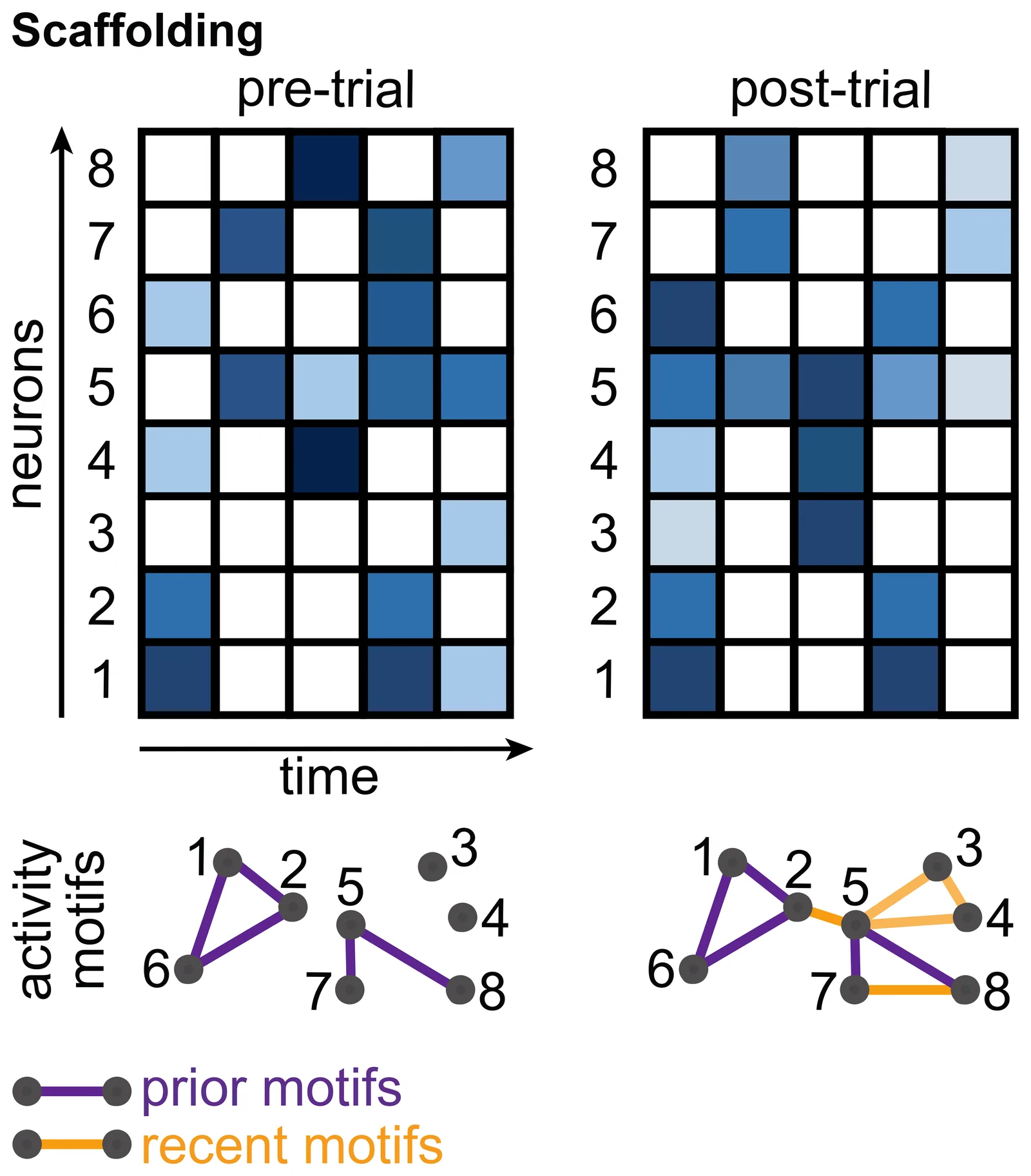
Illustration of representation scaffolding. We illustrate this with cartoons of one population vector (*top*) with each square representing the firing response of a neuron at a given time, before a memory trial (pre-trial) and thereafter (post-trial). We complement this with the corresponding neuronal graph motifs (*bottom*) with purple edges denoting cell pairs constituting prior coactivity motifs (i.e., already present before encoding) while orange edges show coactivity relationships that selectively emerged with memory formation (i.e., absent in the pre-trial epoch).

One important question regards how neural circuits continually learn and integrate new information on top of pre-existing knowledge. This would involve a complex trade-off between stability, plasticity, flexibility, and updatability of population activity patterns. Interestingly, the heterogeneity of hippocampal principal cells gives clues on how this process may work. Higher-activity cells can organize motifs that rapidly discriminate spatial contexts, instantiating a spatio-contextual backbone robust to perturbation by subsequent experience ^165^. Lower-activity cells integrate coactivity motifs on demand, throughout successive experiences, and with a heightened engagement over time to affect pre-existing network representations. A third strategy is therefore representation scaffolding ^9,14,103^ (Figure 7). This is reminiscent of the notion of memory “schema”, referring to an internal framework for effective information processing based on past experiences and knowledge ^65,171–173^. A schema for “birthday party” would allow one to expect certain elements (e.g., a cake with candles) if about to attend one. With this, memories in the works, and to be maintained over the longer term, may rely on core motifs of neuronal activity that instantiate a pre-structured internal scaffold of a stable portion of the population patterns onto which new content can be grafted in another part of the population activity. For example, when repeatedly learning motor tasks that require executing the same action in different contexts, patterns of neural activity reflecting motor cortical memories for task execution combine a fixed part during action execution that is preserved across tasks and a context-dependent part during motor preparation that is orthogonal across contexts ^14^. The hippocampus employs a comparable scaffolding principle during spatial exploration by anchoring a new neural coactivity module (made of lower-firing cells in the CA1 superficial pyramidal sublayer) onto a pre-existing stable module (made of higher-rate cells in the CA1 deep sublayer) ^103,174^.

Finally, complex brain functions require information exchange between multiple brain areas. This distributed communication process could involve a single neuron to send information to several thousand other neurons across different areas. In such a scenario, a fair assumption would be that not all downstream readers may have the same information processing capabilities or be receptive to the same information. A fourth strategy could therefore be that neural codes are designed for information broadcasting to multiple distinct targets. Broadcasting refers to the simultaneous and effective communication of information from a single source to multiple receivers, each possibly receptive to different information contents or with a different capacity to process information. In engineering, how to broadcast information has been well addressed, for example to design channels and codes to transmit television information ^175^. Good broadcast channel codes can give all the important information even to the worst receivers and add more information to the best receivers. Engineering solutions to broadcasting have code features that resemble those observations in neurons, from across-time-scale multiplexing to superposition within the same emitting system of higher-rate and lower-rate information representations ^175^. However, whether and how neural codes are designed to broadcast information remains to be determined.

### Do the same design principles shape neural codes for memory and those for other brain functions?

Memory presents similar neural computations to other brain functions such as sensation or decision-making. It participates in, and partly overlaps with, these other functions. An important question therefore regards whether design principles for neural codes are specific to memory or are shared by different brain functions. For example, codes for sensation and memory could be designed with similar trade-off principles, but they could look different just because the relative importance of competing needs (and thus the relative weight in the objective function) depends strongly on the specific function. Alternatively, neural codes implementing different functions may be designed with entirely different principles.

There is evidence that neural population codes differ across functions or across brain regions implementing primarily different functions. For example, correlations between the activity of hippocampal cells can increase the information content about a spatial position ^150,151^ because they are stronger around the place field, whereas correlations in sensory areas often decrease sensory information ^147^. Neural codes for sensory signals in sensory areas have weaker and short-lived time-lagged correlations that are more suited to encode larger amount of rapidly-varying sensory information, whereas neural codes for choices in association areas have stronger and long-lived time-lagged correlations that are less suited to encode high amounts of information but ensure a better transmission of decision signals to behavioral output and can integrate better information over time ^77,121^. These differences in neural codes across brain functions or regions are in principle compatible with both the above hypotheses. Disambiguating between these hypotheses requires approaches to dissect the nature of trade-offs, which we discuss below.

### How can we empirically determine how neural codes support memory properties, computations and their trade-offs?

Three approaches (which could be employed individually or in combination) can be conceived. The first approach is to manipulate behavioral demands or contingencies to induce changes in how competing needs are traded off and evaluate how features of neural codes respond. For example, the hippocampus uses population activity to support contextual memories, some of which manifest as robust behavioral readouts while others manifest as flexible readouts. By recording hippocampal neurons in mice switching from robust contextual feeding to flexible object recognition within the same environment, recent work illustrates how manipulating the behavioral demand of a task allows identifying the changes in population activity patterns that serve a trade-off between distinct memory properties ^20^. Here, behavioral contingencies creating a robust contextual memory tightly coactivate individual neurons to increase consistency within the neural population and generate a strong behavioral output. Conversely, task contingencies involving the expression of a flexible memory leveraged lower population coupling to allow diverse mnemonic patterns to coexist and drive dynamically adaptable behavior.

A second approach is to assess how perturbing neural codes changes the behavioral ability to cope with competing demands ^15,57,176^. In the study just mentioned, optogenetic silencing the set of hippocampal principal cells selectively recruited in the food-paired context and located in the superficial sublayer of the CA1 pyramidal layer allowed adjusting population coactivity and correlations to lower levels to restore memory flexibility ^20^. This shows that some neural coding features can contribute causally to regulating the trade-off between stability and flexibility. Another study established that optogenetic manipulation of the sequential structure of hippocampal place cells in rats traversing specific spatial trajectories abolished internal replay of these behavioral trajectories and the development of a predictive population code through sequences, thereby impairing learning of new optimal navigation trajectories. This provides evidence that sequences support predictive code ^15^.

The third approach is to leverage data-analytical approaches that exploit naturally occurring (i.e., not driven by an experimental manipulation) trial-by-trial, moment-by-moment, or subject-by-subject changes in features of neural codes to spontaneously occurring changes in the quality of memory task performance ^5,77^. To exemplify it we consider a behavioral task that requires learning and holding in memory an association between a sensory cue and a reward location ^77^. This study used analytical approaches to unmask a trade-off, based on correlation level, between forming an accurate sensory cue representation and generating robust recall and behavior. An analytical trial-shuffling approach showed that the decoding accuracy obtained from pseudo-population vectors generated by shuffling trials to destroy correlations increases cue decoding accuracy compared to the cue decoding accuracy of the real population vectors that contained correlations (Figure 5F). However, correlations were stronger during correct than during incorrect probe trials, indicating that correlations help robust readout of the cue-reward location association. The net result of the competing effects of correlations on the two computations of representation and readout were evaluated by using data-driven behavioral readout models that predict the probability of correct single-trial readout of each neural population patterns. These models predicted that better task performance would be reached with intermediate than with zero correlation values ^77^ (Figure 5E), because these intermediate correlation values better balance the negative effects of correlations on representation accuracy with their positive effects in strengthening readout. As a second example with another neural population feature, subject-by-subject variations in the change of dimensionality between the memory representation of the identity of familiar vs non-familiar individuals correlate with individual performance in social memory tasks, demonstrating the importance of dimensionality and of other aspects of the representational geometry in setting social memory trade-offs ^5,177^. These approaches have been shown to work well to dissect multiple computations and individuate trade-offs in perceptual decision making, evidence accumulation, and social memory tasks.

### Outlook and Conclusions

In this Perspective, we explored the relationship between memory properties, computations, and neural population codes. We discussed how the individual features of neural population codes shape multiple memory properties and computations, benefiting some while hindering others. We thus propose that brain memory circuits undergo dynamic trade-offs between properties with competing benefits and requirements. It remains for future work to document experimentally both the existence of a “bell-shaped” curve balancing trade-offs and whether changes in neural code features reflect new optimal trade-offs under changes in relative importance of competing needs. For example, at one end, a reduced coactivity could correspond to weaker memory expression but greater memory flexibility; at the other end, higher coactivity levels could reflect powerful memories but reduced flexibility. Short sequences (with a high compression of time) could serve memory consolidation by allowing more replies in shorter epochs; longer sequences may help maintaining steady memory content over time, which may e.g. aid in implementing behaviors, such as goal-directed navigation, that require consistent information over longer time scales^121^. Yet, we propose possible neural solutions for population trade-offs, such as setting values of code features to optimally balance competing demands or parallelizing processing channels across space (e.g., brain regions) and time. Population trade-offs may create a “safe zone” where a memory circuit operates in the most adaptive (optimized) manner. Dysfunctional memories exist along a broad spectrum, ranging from weakened expression strength to excessively heightened expression. Identifying such safe zone within the population activity space is an important challenge for both discovery and translational neuroscience of memory. This could leverage innovative combinations of behavioral, neural, and analytical approaches.

We anticipate that this effort would gain from systematically linking features of population spiking activity with features of network-level activity patterns. A considerable amount of work has emphasized the importance of network rhythms (e.g., theta oscillations) and burst events (e.g., sharp-wave/ripples). Across the large memory research field, this mesoscopic aspect of neural activity has not always been integrated with the study of multiple single-neuron spiking codes. Future research faces the challenge of integrating information from these two organizational levels and uncovering what might be overlooked by not connecting them. Along this line, it is increasingly recognized that a given network pattern can exhibit some inhomogeneities (e.g., in the spatial, frequency, or time domains). These can provide important information regarding how a memory circuit handles trade-offs. For instance, theta-band oscillations are related to the neural syntax for memory, segmenting population activity vectors for better representation or transmission ^38^. Theta oscillations provide windows for local computations ^178^ that may, for example, allow transient expression of internal models to predict neural inputs and update representations by comparing a top-down prediction and a bottom-up input ^179^. Studying cycle-by-cycle variability of theta oscillations using their higher-frequency contents has indicated multiplexing for population-level trading off between encoding and retrieval ^122,168^. Likewise, diversity of hippocampal ripples may also reflects circuit-level trade-offs ^16,17,62,103^. Oscillatory coordination of brain distributed neuronal spiking can increase or dynamically vary the strength of correlations across population firing vectors, thereby adding flexibility to the operations of memory codes. Neural oscillations can support a holistic integration of population codes distributed over brain networks that are not directly connected. By exploring side-by-side network oscillations and population spiking, work showed that short bouts of higher-power beta-band oscillations coordinated across brain regions by the phase of a slower (4-Hz) rhythm are related to temporal correlation of distributed neuronal spiking, in association with robust drug-paired memory expression ^126^. Beta-frequency oscillations report novelty detection under normal conditions ^180^. This oscillatory structure seems to be compromised in a mouse model of Alzheimer’s disease showing weak memory ^181^. These results illustrate the proposed idea of a bell-shaped curve where changes in the relationships between the value of a given network-level activity variable (e.g., power) and that of a population activity feature (e.g., correlation) relate to memory expression strength.

As technology advances, enabling higher-density simultaneous recording of both LFPs and single-cell spiking activity from larger populations of neurons across behavioral states and contingencies, further developments of conceptual frameworks is essential. These frameworks will help interpret the data and characterize how different features and spatio-temporal scales of neural activity influence each other. We see it as a key challenge to determine what information is uniquely carried by each neural population feature of level of activity organization, and what information processing is instead synergistically created by different neural population features or activity organized at multiple levels. In unperturbed conditions, population code features or activity at different levels are often coupled. For example, strong zero-lag correlations may reduce the dimensionality of neural representations, tuning diversity may enhance the dimensionality, and across-neuron correlations may influence LFP power. These relationships may make it difficult to disentangle their unique contribution to memory function and to trade-offs. To enable testing their unique roles in brain functions, it will thus be essential to develop perturbation techniques that can decouple features of population codes and activity at different levels.

Addressing these challenges could lead to major progress in understanding the neural population computations and codes for memory, and how their dynamic organization supports the multifaceted nature of this critical brain function.

## Glossary

### Abbreviations

**Properties of memory patterns**

Veridicality: the property to truthfully reflect the world
Storability: the property to be retained over time
Accessibility: the property to be readily reached and retrieved from storage
Efficiency: the property to operate effectively with limited metabolic expenditure
Stability: the property to resist changes and disruptions, including passage of time
Accuracy: the property to be reliably decoded when reinstated
Plasticity: the property to undergo an enduring change for a transformative adaptation in response to an external influence
Flexibility: the property to undergo a transient adjustment for an immediate adaptation in response to an external influence
Updatability: the property to undergo an internal improvement process enhancing functionality by e.g. editing past information or integrating new information
Rheostasis: the adjustment of the internal conditions of a biological system to maintain functional effectiveness when external conditions change
Generalizability: the property to represent information robustly across distinct conditions beyond the original one
Relationality: the property to establish relationships with other neural patterns
Predictivity: the property to allow forecasting future events and outcomes based on currently held information
Readability: the property to be effectively read out and transmitted downstream

**Features of population codes**

Sparsity: the feature that reports the fraction of neurons recruited at a given time
Coactivity: the feature that refers to the short-timescale coincidental firing of multiple neurons
Geometry: the set of features that describes the geometrical organization of the population activity space and includes its embedding dimensionality and curvature
Correlation: the feature that describes the covariations across trials of firing of different neurons (across-neuron correlations) or of population vectors at different times (across-time correlations)
Heterogeneity: the feature that relates to the diversity of neuronal responses
Sequentiality: the feature that reports multiple neurons firing in a specific order

**Strategies of population coding**

Optimal balancing: a strategy for trading off competing demands by optimally weighting the relative cost of each simultaneously engaged memory operation
Objective function: a mathematical function defining the goal of an optimization problem, guiding the selection of variable values to maximize or minimize a specific objective
Parallel processing: a strategy for resolving competing memory demands by assigning specific computations to different neural units
Representation scaffolding: a strategy for continually learning and integrating new information on top of a neural backbone of pre-existing knowledge
Information broadcasting: a strategy to design population codes that can communicate effectively information simultaneously to many distinct receivers with distinct properties
